# Comparative Ultrasonographic Evaluation of Morphology and Vascularization in Endometriomas and Ovarian Mature Cystic Teratomas †

**DOI:** 10.3390/jcm14196912

**Published:** 2025-09-29

**Authors:** Aleksandar Rakić, Elena Đaković, Zagorka Milovanović, Aleksandar Ristić, Lazar Nejković, Ana Đorđević, Jelena Brakus, Jelena Štulić, Žaklina Jurišić, Aleksandar Jurišić

**Affiliations:** 1 Obstetrics and Gynecology Clinic Narodni Front, Kraljice Natalije 62, 11000 Belgrade, Serbia; a.r.rakic@gmail.com (A.R.); djakovicelena7@gmail.com (E.Đ.); zaga_mil@yahoo.co.uk (Z.M.); ristic.aleksandar@icloud.com (A.R.); lnejkovic@sbb.rs (L.N.); ankec96@gmail.com (A.Đ.); jelena1st@yahoo.com (J.B.); jstulicbgd@gmail.com (J.Š.); 2 School of Medicine, University of Belgrade, 11000 Belgrade, Serbia; 3 Obsetrics and Gynecology Polyclinic Jurisic, 11000 Belgrade, Serbia; zaklina@jurisicordinacija.rs

**Keywords:** endometrioma, mature cystic teratoma, ultrasonography, morphology, vascularization, Doppler

## Abstract

**Background/Objectives**: Adnexal masses are commonly encountered in the routine practice of gynecologists, and transvaginal ultrasonography is the preferred imaging modality for assessing the masses in size and complexity. There has been a notable lack of focus on comparative studies concerning benign adnexal tumors. This study aimed to define and compare the specific morphological and vascular characteristics of ovarian mature cystic teratomas (MCTs) and endometriomas using transvaginal ultrasound and Doppler analysis. **Methods**: This retrospective analysis included 93 patients who underwent surgical intervention for benign adnexal masses at the Obstetrics and Gynecology Clinic Narodni Front from 1 January 2020 to 1 January 2022. Morphological parameters included the appearance of tumors, the largest diameter, volume, capsule thickness, and the presence of fluid in the pouch of Douglas. Hemodynamic parameters included the localization and quantity of blood vessels within the mass, Resistance Index (RI), peak systolic velocity (Vmax), and end-diastolic velocity (Vmin) within detectable tumor vessels. Flow was also assessed in the uterine arteries, calculating the AURI (uterine artery RI) on both the tumor and contralateral sides. **Results**: There were 46 patients with ovarian mature cystic teratomas, as well as 46 patients with endometriomas; 1 patient presented with both tumors. There were significant differences in ultrasonographic morphological appearance between the two groups. MCTs most frequently presented as multilocular solid cysts (51.0%) or unilocular solid cysts with hyperechoic content (20.4%). Conversely, the majority of endometriomas were classified as unilocular cysts with ground-glass echogenicity (45.5%). A significant difference was identified in the RI of intracystic vessels and the RI of the ipsilateral uterine artery (AURI). Endometriomas presented elevated RI values (0.57 vs. 0.54, *p* = 0.04) and reduced AURI (0.81 vs. 0.83, *p* = 0.02) compared to teratomas. **Conclusions**: These findings confirm that specific morphological and Doppler parameters, particularly the RI and AURI, can assist in distinguishing between endometriomas and mature cystic teratomas. This suggests a potential role for Doppler analysis in improving diagnostic precision for common benign adnexal tumors in clinical practice.

## 1. Introduction

Adnexal masses are a common clinical finding in routine gynecological practice, with prevalence and incidence rates demonstrating significant variation by age and reproductive status. In the general premenopausal female population, these masses are frequently encountered, with an estimated 6–8% of women affected [1]. The incidence among women of reproductive age rises dramatically from childhood, increasing from a minimal rate in young girls to a peak of approximately 152 per 100,000 women by the age of 35 [2]. Moreover, they are identified in up to 8.8% of pregnancies [3]. There is also a substantial lifetime surgical burden, as up to 10% of all women will undergo surgery for an adnexal mass [4]. Further factors associated with the prevalence of adnexal masses include the characteristics of the masses and the presence of symptoms associated with these masses.

Transvaginal ultrasonography (TVUS) is the preferred imaging modality for assessing adnexal masses in size and complexity [4]. Many investigations have attempted to identify ultrasonographic characteristics that enhance diagnostic precision and guide surgical choices regarding malignancy risk. This effort has led to the development of highly accurate evidence-based risk stratification systems, such as the International Ovarian Tumor Analysis (IOTA) group’s Simple Rules, the ADNEX model, and the Ovarian-Adnexal Reporting and Data System (O-RADS) [5,6]. These models demonstrate impressive accuracy in preoperative differentiation between benign and malignant adnexal masses [5,6], but they are not specifically designed to discriminate between different types of benign tumors. Consequently, there has been a notable lack of focus on comparative studies concerning benign adnexal tumors.

In most clinical situations, the combination of IOTA-based models and expert pattern recognition provides sufficient diagnostic accuracy. Endometriomas, for example, are typically recognized as unilocular cysts with ground-glass echogenicity and without solid components, while mature cystic teratomas (dermoid cysts) are usually identified by the presence of Rokitansky nodules, echogenic sebaceous material, or hair–fluid levels [7,8,9]. However, clinical practice also frequently presents cases in which the distinction between these two common benign tumors is not straightforward. Endometriomas may sometimes appear multilocular, heterogeneous, or with solid-appearing papillary structures, whereas dermoid cysts may lack their classical echogenic features or mimic hemorrhagic corpus luteum cysts [10,11]. Such atypical presentations are not uncommon and can cause diagnostic difficulties, especially among less experienced sonographers. Importantly, existing IOTA and O-RADS frameworks are not specifically designed to address differentiation between benign subtypes, but rather to stratify the risk of malignancy. This leaves a gap in standardized guidance for cases where the main diagnostic challenge lies between two benign entities.

On the other hand, knowledge of the role of vascularization in the ultrasonographic assessment of benign adnexal tumors remains limited. Vascular indices, such as the Resistance Index (RI) or the uterine artery Resistance Index (AURI), have been evaluated in attempts to differentiate malignant from benign masses. The role of these indices in distinguishing between different benign subtypes remains limited [12,13]. Hemodynamic parameters could potentially reflect different biological behaviors and features of endometriomas and dermoids, resulting in different Doppler patterns. While these indices are not included in the existing malignancy prediction models, exploring them in benign scenarios might provide additional insights and may be especially beneficial in atypical cases, where morphology alone is unreliable.

Therefore, the primary aim of this study was to perform a direct, detailed comparison of the morphological and, more importantly, hemodynamic characteristics of ovarian endometriomas and mature cystic teratomas, as assessed by TVUS. We sought to determine whether Doppler parameters, specifically RI and AURI, could provide objective criteria to supplement morphological assessment and improve diagnostic confidence in differentiating these two common benign conditions.

## 2. Materials and Methods

### 2.1. Study Design and Participants

This retrospective study included patients who underwent surgical treatment for benign adnexal masses at the Obstetrics and Gynecology Clinic Narodni Front from 1 January 2020 to 1 January 2022. Ethical review and approval were waived for this study by the ethics committee due to its retrospective design, which involved the analysis of anonymized pre-existing clinical data obtained as part of standard diagnostic care. This waiver is in accordance with national regulations and the institution’s policy on retrospective studies.

Surgical intervention was indicated based on the following factors: the size of the adnexal mass (an institutional threshold for surgery in asymptomatic patients), persistent pelvic pain attributable to the mass, or an inconclusive sonographic examination where the features were ambiguous and did not allow for a definitive diagnosis of a classic benign lesion.

The inclusion criteria for the study were as follows: (1) preoperative transvaginal ultrasonography performed at our clinic, and (2) a confirmed histopathological diagnosis of either ovarian endometrioma or mature cystic teratoma.

The exclusion criteria were as follows: (1) insufficient ultrasound image quality for retrospective analysis, (2) pregnancy at the time of the scan, (3) a final histological diagnosis other than endometrioma or MCT, or (4) medical treatment (e.g., hormonal therapy) in the 3 months preceding the ultrasound.

### 2.2. Ultrasonographic Examination and Data Analysis

All TVUS examinations were performed by experienced gynecologists using a Voluson E10 system (GE Healthcare, Boston, MA, USA) with a high-resolution intracavitary probe (RIC5-9-D, 5–9 MHz).

For this study, stored ultrasound images and cine loops were retrospectively and independently analyzed by two experienced sonographers (E.Đ. and A.J.). To minimize assessment bias, the reviewers were blinded to the patients’ final histological diagnosis and clinical symptoms during the offline analysis.

The sonographic features of all masses were described according to the terminology and definitions established by the International Ovarian Tumor Analysis (IOTA) group [5].

Two types of features were analyzed:

1. Morphological Parameters: The morphological assessment included the largest tumor diameter, volume (calculated using the prolate ellipsoid formula: length × height × width × 0.523), laterality, capsule thickness, and internal echogenicity (categorized as anechoic, homogeneous low-level “ground-glass”, hyperechoic, or mixed/heterogeneous). The presence of septa, papillary projections, solid components, acoustic shadowing, free fluid in the pouch of Douglas, and signs of tumor fixation were also recorded.
2. Hemodynamic Parameters: Assessed using color and pulsed Doppler techniques. The location of blood flow (pericystic, intracystic, in septa) was noted. The peak systolic velocity (Vmax), end-diastolic velocity (Vmin), and Resistance Index (RI) were measured from spectral Doppler waveforms. Measurements were also taken from the ipsilateral uterine artery to obtain its Resistance Index (AURI).

The definitive diagnosis was established by histopathological examination of the surgical specimens, which served as the gold standard.

### 2.3. Statistical Analysis

Descriptive statistics were reported as mean values accompanied by standard deviations (SDs) for numerical variables, or as absolute counts with percentages for categorical variables. The distinctions between the two groups were presented as mean differences accompanied by 95% confidence intervals and analyzed using Student’s *t*-test for numerical data and chi-squared tests for categorical data. In all assessments, a *p*-value of less than 0.05 indicated statistical significance. Statistical analysis was conducted using IBM SPSS statistical software (SPSS for Windows, release 25.0, SPSS, Chicago, IL, USA).

## 3. Results

This study included a total of 93 patients. Based on histopathological examination, 47 patients were diagnosed with endometriomas, and 47 patients were diagnosed with MCTs. One patient was diagnosed with both tumors synchronously. The demographic and reproductive characteristics of the patients are presented in Table 1. There were no significant differences in age between the two groups (37.8 ± 10.2 vs. 38.4 ± 13.3 years, *p* = 0.8). However, the teratoma group had a significantly higher parity rate (median 1 vs. 0, *p* = 0.02). The cohort included 16 postmenopausal women (12.8% of the endometrioma group and 23.4% of the teratoma group, *p* = 0.2).

There were no significant differences between the groups in tumor size, volume, capsule thickness, septal thickness, the presence of free fluid, or tumor fixation (Table 2). There was a significant difference in tumor laterality, with endometriomas being more frequently bilateral than teratomas (17.0% vs. 4.3%, *p* = 0.042).

There were significant differences in the ultrasonographic morphological appearance between the two groups classified according to the IOTA terminology (*p* < 0.001, Table 3). MCTs most frequently presented as multilocular solid cysts (51.0%) or unilocular solid cysts with hyperechoic content (20.4%). On the other hand, the majority of endometriomas were classified as unilocular cysts with ground-glass echogenicity (45.5%). Other morphological types were observed less frequently in both groups.

**Figure 1:**
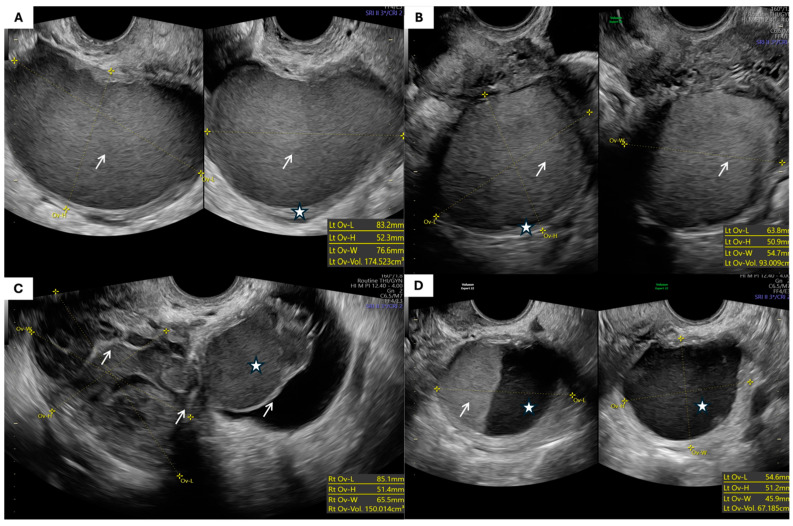
Ultrasonographic appearances of endometriomas: (**A**,**B**) Unilocular cyst with ground-glass echogenicity (arrows indicate characteristic ground-glass echogenic content; asterisks mark the thin cyst wall). (**C**) Multilocular endometrioma (arrows point to septations; asterisk marks the largest locule with ground-glass echogenicity). (**D**) Multilocular solid endometrioma (arrows indicate solid components and septations; asterisk marks cystic component with ground-glass appearance).

**Figure 2:**
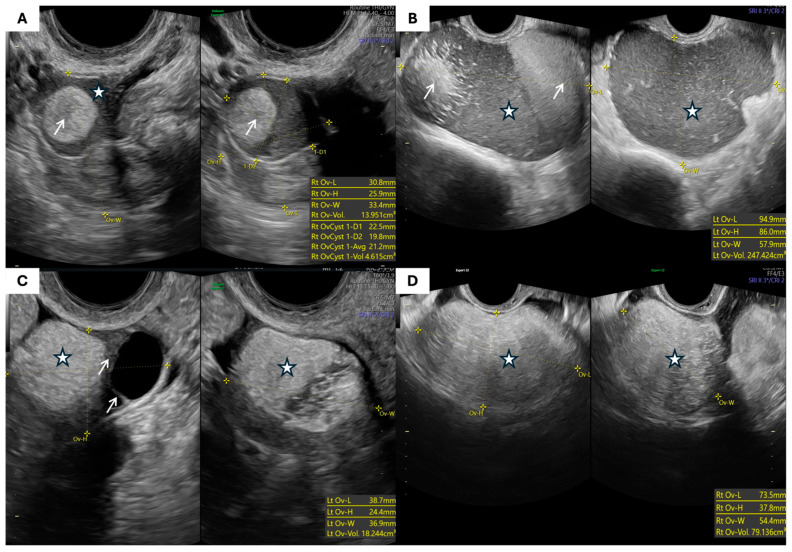
Ultrasonographic appearances of mature cystic teratomas: (**A**,**B**) Multilocular solid ovarian teratoma (arrows point to hyperechoic areas and solid tissue components; asterisks mark cystic components). (**C**) Multilocular solid ovarian teratoma (arrows point to septations; asterisk indicates hyperechoic solid component). (**D**) Unilocular solid ovarian teratoma (asterisk indicates hyperechoic solid component).

The most common morphological types of endometriomas and ovarian teratomas are presented in Figure 1 and Figure 2, respectively.

**Figure 3:**
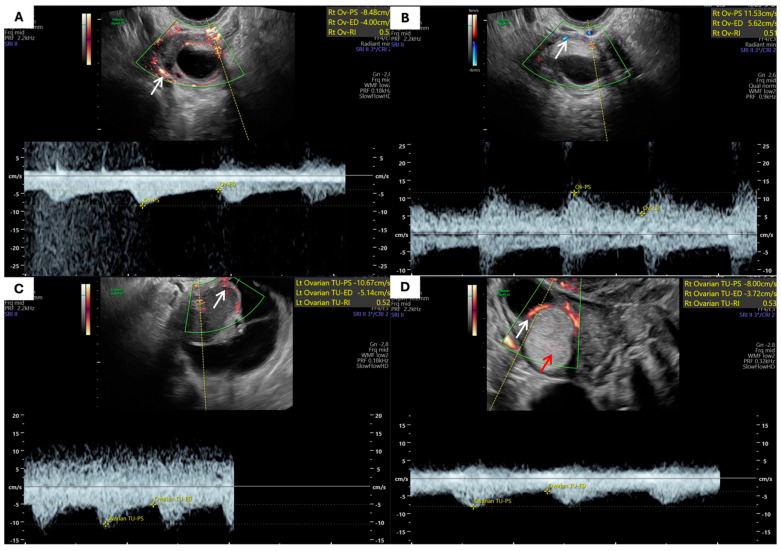
Examples of vascularization patterns: (**A**,**B**) Pericystic vascularization in ovarian mature teratoma (arrows indicate pericystic blood vessels). (**C**) Vessels within the septum of an endometrioma (arrows point to vascular signals within the septum). (**D**) Vascular pattern in a multicomponent ovarian teratoma (white arrows highlight blood vessels within the septa; red arrow points to a vascular signal within the solid component).

The overall vascularization pattern did not differ significantly between the groups. The total number of blood vessels was slightly higher in endometriomas (n = 168) compared to MCTs (n = 149), but the mean number of blood vessels was comparable (3.03 ± 1.67 vs. 3.00 ± 2.30, *p* = 0.5). The most frequent localization of blood vessels in both groups was pericystic, followed by vessels in septa, solid regions, or multiple locations, with no statistically significant differences (*p* = 0.5). Vascularization patterns, along with blood vessel features, are presented in Table 4. Examples of blood vessel localizations are presented in Figure 3.

A significant difference was identified in the Resistance Index (RI) and AURI of the impacted side, with endometriomas presenting elevated RI values (0.57 vs. 0.54, *p* = 0.04) and reduced AURI (0.81 vs. 0.83, *p* = 0.02) when compared with teratomas. Additional Doppler parameters, such as Vmax, Vmin, and vascular asymmetry indices (ΔAURI and ΔVmaxAU), exhibited no significant differences between the two groups (Table 5).

## 4. Discussion

The results of our study revealed significant differences in both morphological and hemodynamic characteristics between endometriomas and MCTs. Morphologically, endometriomas more frequently appeared as unilocular cysts with homogeneous low-level echogenicity, while MCTs typically manifested as cystic lesions with solid components or heterogeneous echogenicity. More importantly, our Doppler analysis further identified hemodynamic differences: a significant difference was observed in the RI and AURI of the impacted side, with endometriomas presenting elevated RI values (0.57 vs. 0.54, *p* = 0.04) and reduced AURI (0.81 vs. 0.83, *p* = 0.02) compared to MCTs. Additional Doppler parameters showed no significant differences between the two groups.

Our findings strongly support the established sonographic patterns associated with endometriomas and MCTs. MCTs typically present with distinctive ultrasonographic characteristics, often including “dots and/or lines” and an “echogenic white ball” [14,15]. Conversely, an endometrioma is generally unilocular, with low-level “ground-glass” echogenicity [7]. Although endometriotic implants may show cyclical changes, ovarian endometriomas typically retain a stable sonographic appearance throughout the menstrual cycle. This stability is useful in clinical differentiation from hemorrhagic corpus luteum cysts, which may undergo rapid morphological changes over short intervals [16].

The vascularization of adnexal masses represents a cornerstone of sonographic assessment, primarily utilized in established models like the IOTA Simple Rules to stratify malignancy risk by distinguishing avascular (Score 1) from highly vascularized lesions (Score 4) [5,17]. This paradigm is grounded in the understanding that malignant tumors provoke intense neovascularization to facilitate their rapid growth. This leads to the formation of vessels that are both structurally and functionally compromised, as shown by high density yet low impedance. This is evident sonographically as low RI values [18,19,20,21]. Our study confirms that both endometriomas and MCTs align with benign vascular patterns, exhibiting primarily pericystic vascularization without the marked neovascularization characteristic of malignancies.

Our findings demonstrate that within the benign spectrum, there are notable and mechanistically informative hemodynamic differences. The higher intratumoral RI in endometriomas presents a vascular paradox. While endometriosis is a state of heightened angiogenic activity, it results in chaotic and dysfunctional vasculature. Endometriotic lesions are characterized by estrogen-dependent angiogenesis and a profound inflammatory microenvironment, marked by elevated TNF-α and interleukins, which promote persistent endothelial cell activation and neovascularization [22]. Crucially, ovarian endometriomas exhibit elevated HIF-1α levels compared to normal endometrium. Chronic bleeding, iron deposition, and subsequent tissue hypoxia drive sustained overexpression of VEGF and lead to pathological angiogenesis [23,24]. However, the process results in immature vessels with incomplete pericyte coverage and significant surrounding fibrosis. This disorganization leads to an increase in vascular impedance and a decrease in diastolic flow, which are sonographically represented by the elevated RI that we noted.

Conversely, the lower RI in teratomas reflects a different, more organized vascularization pattern, which could be a result of their developmental origin. Since MCTs are formed by tissues from three germ layers [25], their heterogeneous but well-differentiated components (neural, adipose, and epithelial) create different microenvironments that promote developmental angiogenesis. This process typically results in more organized vascular networks that reflect the developmental origin of the heterogeneous tissue components [26]. These characteristics lead to lower resistance to blood flow and the lower RI values that we recorded.

The reduced AURI on the side affected by endometriomas indicates a more extensive pelvic hemodynamic influence, potentially representing compensatory vasodilation induced by the inflammatory cascade associated with endometriosis [27,28,29]. This indicates a widespread hemodynamic adaptation that is absent in the isolated, non-inflammatory teratomas.

While RI and AURI are not incorporated into current IOTA or O-RADS models, which are exclusively focused on malignancy risk stratification, our results suggest that these quantitative Doppler parameters could have significant potential for refining the differentiation between common benign tumors. The combination of a higher intratumoral RI and a lower ipsilateral AURI could form a distinctive “hemodynamic signature” more suggestive of an endometrioma. In practical settings, especially those with restricted access to expert sonography or advanced modeling, this objective hemodynamic assessment could enhance morphological evaluation and facilitate more informed decision-making in clinically ambiguous cases. Moreover, RI and AURI values offer standardized, numerical metrics that minimize dependence on subjective interpretation, ensuring consistent reproducibility across various operators and clinical settings. Objective measurements enhance communication among healthcare providers and promote more consistent reporting. Understanding the various morphological characteristics and vascularization patterns can benefit gynecologists in their everyday clinical work. Furthermore, our findings could have implications for educational applications. They provide a systematic framework for experienced clinicians to instruct inexperienced medical professionals in identifying distinctive sonographic patterns of common benign adnexal tumors. This pattern-based interpretative approach may increase diagnostic confidence in both training and routine practice.

On the other hand, this study has several limitations. First of all, the retrospective, single-center design presents an inherent risk of selection bias, as it exclusively included patients who underwent surgical intervention, likely reflecting a group with more symptomatic or complex masses. The small sample size of postmenopausal women (n = 16) in our cohort prevented a robust statistical analysis regarding the possible confounding influence of menopausal status on our Doppler parameters. This is an important factor to consider, as the sonographic features of an atypical appearance of adnexal mass in postmenopausal women require careful evaluation due to the significant risk of malignancy linked to these lesions in this population [30]. While the analysis was conducted by skilled sonographers, the lack of formal assessment for inter-observer agreement regarding morphological classification presents a notable methodological limitation. Additionally, we were unable to record tumor marker values (e.g., CA-125) for every patient. The incorporation of this data would have offered significant correlative insights and enriched the clinical context surrounding our ultrasonographic findings. Additional potential confounding factors, including the phase of the menstrual cycle, were not examined and require consideration in future research.

## 5. Conclusions

The results of our study not only confirm IOTA recognition patterns as the gold standard in the differentiation between endometriomas and mature cystic teratomas but also indicate a significant difference in the vascular pattern between these two benign adnexal masses. Elevated RI within the tumor, with significantly lower AURI on the ipsilateral side in endometriomas, may indicate a different hemodynamic signature between the two tumor types: a more chaotic vascular network fueled by complex inflammatory processes and a more stable and scarce vascularization of the teratoma, most likely as a consequence of a specific embryonic origin. While current risk stratification models focus solely on malignancy, these objective Doppler parameters have a promising potential as a valuable addition to B-mode ultrasound. By providing reproducible numerical data, they might strengthen diagnostic confidence in ambiguous cases and improve differential diagnosis, ultimately facilitating more accurate clinical decision-making. However, for the validation of these results and investigation in order to incorporate specific Doppler parameters into existing predictive models, and to determine how exactly a different vascular pattern would help in differentiating between these two types of benign adnexal tumors, it is necessary to conduct larger, multicenter studies with experienced ultrasonographic experts.

## Figures and Tables

**Table 1:** Demographic and reproductive features of patients with endometrioma and ovarian mature cystic teratoma.

Parameter	Patients with Endometrioma (n = 47)	Patients with MCT (n = 47)	*p*-Value
Age (years), mean ± SD	37.8 ± 10.2	38.4 ± 13.3	0.8
Menopausal status, n (%)			0.2
Premenopausal	41 (87.2%)	36 (76.6%)	
Menopausal	6 (12.8%)	11 (23.4%)	
Duration of menopause (years), mean ± SD	2.0 ± 2.9 *	8.1 ± 9.5 **	0.1
Parity, median [IQR]	0 [0–1]	1 [0–2]	0.02 ***

* Data presented for 6 postmenopausal patients with endometrioma. ** Data presented for 11 postmenopausal patients with teratoma; *p*-values for menopausal status variables calculated using Fisher’s exact test; *p*-value for parity of menopause calculated using the Mann–Whitney U test; *p*-value for age and duration of menopause calculated using Student’s *t*-test; *** indicates a statistically significant difference.

**Table 2:** Ultrasonographic characteristics of tumors in patients with endometriomas and ovarian teratomas.

Parameter	Endometriomas (n = 47)	MCTs (n = 47)	*p*-Value
The largest tumor dimension (mm), mean ± SD	78.2 ± 3.3	81.9 ± 4.3	0.6
Tumor volume (cm^3^), mean ± SD	247.9 ± 65.2	254.3 ± 75.3	0.7
Tumor capsule thickness (mm), mean ± SD	2.87 ± 1.1	2.9 ± 1.1	0.9
Septal thickness (mm), mean ± SD	2.6 ± 0.7	2.7 ± 1.4	0.7
Free fluid in the pouch of Douglas, n (%)	1 (2.1%)	2 (4.3%)	0.500
Tumor fixation **, n (%)	7 (14.9%)	3 (6.4%)	0.200
Tumor localization, n (%)			0.042 *
Unilateral	39 (83.0%)	45 (95.7%)	
Bilateral	8 (17.0%)	2 (4.3%)	

Note: *p*-values for categorical variables (free fluid, tumor fixation, tumor localization) calculated using Fisher’s exact test. * Statistically significant difference. ** Adhesions between the tumor and surrounding structures.

**Table 3:** Morphological classification of tumors based on IOTA terminology.

Morphological Type (IOTA Terminology)	Endometriomas (n = 55)	%	Teratomas (n = 49)	%	*p*-Value
Unilocular cyst with ground-glass echogenicity	25	45.5%	0	0%	<0.001 *
Unilocular solid cyst with hyperechoic content	1	1.8%	10	20.4%	
Multilocular cyst	8	14.5%	5	10.2%	
Multilocular solid cyst	9	16.4%	25	51.0%	
Cyst with papillary projections (unilocular or bilocular)	1	1.8%	1	2.0%	
Solid tumor	3	5.5%	4	8.2%	
Other/not otherwise specified	8	14.5%	4	8.2%	

* Statistically significant difference; *p*-value calculated using chi-squared test for the overall distribution of morphological types between groups.

**Table 4:** Vascularization characteristics in endometriomas and ovarian teratomas.

Parameter	Endometriomas	MCTs	*p*-Value
Total number of analyzed blood vessels (across all tumors)	168	149	—
Mean number of blood vessels (n ± SD)	3.03 ± 1.67	3.00 ± 2.30	0.5
Localization of blood vessels (n)			0.5
– Pericystic	125	111	
– In septa	24	20	
– Within the papillary projection	6	2	
– Within the solid-appearing components *	13	16	

* Defined as non-fluid, non-fat, and non-calcified tissue.

**Table 5:** Doppler parameters in endometriomas and ovarian teratomas.

Parameter (n ± SD)	Endometriomas	Ovarian Teratomas	*p*-Value
RI	0.57 ± 0.11	0.54 ± 0.13	0.04 *
Vmax	10.72 ± 5.56	10.28 ± 5.96	0.5
Vmin	4.43 ± 2.24	4.34 ± 2.20	0.7
AURI tumor	0.81 ± 0.14	0.83 ± 0.07	0.02 *
AURI contralateral	0.84 ± 0.07	0.85 ± 0.07	0.7
ΔAURI	0.03 ± 0.11	0.01 ± 0.07	0.8
AUVmax tumor	42.62 ± 21.73	38.10 ± 15.0	0.2
AUVmax contralateral	36.80 ± 17.27	34.50 ± 13.45	0.5
ΔVmaxAU	4.73 ± 24.28	3.23 ± 14.88	0.6

* Statistically significant difference; AU—uterine artery; RI—Resistance Index; Vmax—maximal systolic flow velocity; Vmin—minimal diastolic flow velocity; AURI—Resistance Index in uterine arteries; ΔAURI—difference in uterine artery RI between the tumor side and the contralateral side; AUVmax—Maximal systolic flow velocity in uterine arteries; ΔVmaxAU—difference in uterine artery Vmax between the tumor side and the contralateral side.

## Data Availability

The data supporting the findings of this study are available from the corresponding author upon reasonable request.

## Abbreviations

ANDEX	Adnexal Mass Evaluation System
AU	Uterine Artery
AURI	Resistance Index in Uterine Arteries
ΔAURI	Difference in AURI between Tumor and Contralateral Sides
ΔVmaxAU	Difference in Uterine Artery Vmax between Tumor and Contralateral Sides
Doppler RI	Doppler Resistance Index
FGF	Fibroblast Growth Factor
HIF-1A	Hypoxia-Inducible Factor 1 Alpha
IOTA	International Ovarian Tumor Analysis
MCT	Mature Cystic Teratoma
MIF	Macrophage Migration Inhibitory Factor
O-RADS	Ovarian-Adnexal Reporting and Data System
RI	Resistance Index
SD	Standard Deviation
SPSS	Statistical Package for the Social Sciences
sVEGFR-2	Soluble Vascular Endothelial Growth Factor Receptor-2
TVUS	Transvaginal Ultrasonography
VEGF	Vascular Endothelial Growth Factor
Vmax	Maximal Systolic Flow Velocity
Vmin	Minimal Diastolic Flow Velocity
